# Supplementary material for: A fault-intrusion-tolerant system and deadline-aware algorithm for scheduling scientific workflow in the cloud
Source: PeerJ Comput Sci. 2021 Nov 2;7:e747. doi: 10.7717/peerj-cs.747 (PMC8576554; doi:10.7717/peerj-cs.747)
Supplement: Supplemental Information 6 [file peerj-cs-07-747-s006.zip › Myproject1.1/docs/org/cloudbus/cloudsim/network/datacenter/NetworkCloudlet.html]

NetworkCloudlet


JavaScript is disabled on your browser.


- Overview
- Package
- Class
- Use
- Tree
- Deprecated
- Index
- Help

- Prev Class
- Next Class

- Frames
- No Frames

- All Classes

- Summary:
- Nested |
- Field |
- Constr |
- Method

- Detail:
- Field |
- Constr |
- Method


org.cloudbus.cloudsim.network.datacenter

## Class NetworkCloudlet

- java.lang.Object
- - org.cloudbus.cloudsim.Cloudlet
  - - org.cloudbus.cloudsim.network.datacenter.NetworkCloudlet

- All Implemented Interfaces:
  :   java.lang.Comparable<java.lang.Object>

  ---

    

  ```
  public class NetworkCloudlet
  extends Cloudlet
  implements java.lang.Comparable<java.lang.Object>
  ```

  NetworkCloudlet class extends Cloudlet to support simulation of complex applications. Each such
  network Cloudlet represents a task of the application. Each task consists of several stages.
  Please refer to following publication for more details:
  Saurabh Kumar Garg and Rajkumar Buyya, NetworkCloudSim: Modelling Parallel Applications in Cloud
  Simulations, Proceedings of the 4th IEEE/ACM International Conference on Utility and Cloud
  Computing (UCC 2011, IEEE CS Press, USA), Melbourne, Australia, December 5-7, 2011.

  Since:
  :   CloudSim Toolkit 1.0

- - ### Field Summary

    Fields

    | Modifier and Type | Field and Description |
    | --- | --- |
    | `int` | `currStagenum` |
    | `double` | `exetime` |
    | `double` | `finishtime` |
    | `double` | `numStage` |
    | `java.util.ArrayList<TaskStage>` | `stages` |
    | `double` | `starttime` |
    | `double` | `submittime` |
    | `java.util.Map<java.lang.Double,HostPacket>` | `timeCommunicate` |
    | `double` | `timespentInStage` |
    | `double` | `timetostartStage` |

    - ### Fields inherited from class org.cloudbus.cloudsim.Cloudlet

      `accumulatedBwCost, CANCELED, costPerBw, CREATED, FAILED, FAILED_RESOURCE_UNAVAILABLE, INEXEC, PAUSED, QUEUED, READY, RESUMED, SUCCESS, vmId`
  - ### Constructor Summary

    Constructors

    Constructor and Description

    `NetworkCloudlet(int cloudletId,
    long cloudletLength,
    int pesNumber,
    long cloudletFileSize,
    long cloudletOutputSize,
    long memory,
    UtilizationModel utilizationModelCpu,
    UtilizationModel utilizationModelRam,
    UtilizationModel utilizationModelBw)`
  - ### Method Summary

    Methods

    | Modifier and Type | Method and Description |
    | --- | --- |
    | `int` | `compareTo(java.lang.Object arg0)` |
    | `double` | `getSubmittime()` |

    - ### Methods inherited from class org.cloudbus.cloudsim.Cloudlet

      `addRequiredFile, deleteRequiredFile, getActualCPUTime, getActualCPUTime, getAllResourceId, getAllResourceName, getClassType, getCloudletFileSize, getCloudletFinishedSoFar, getCloudletFinishedSoFar, getCloudletHistory, getCloudletId, getCloudletLength, getCloudletOutputSize, getCloudletStatus, getCloudletStatusString, getCloudletTotalLength, getCostPerSec, getCostPerSec, getExecStartTime, getFinishTime, getNetServiceLevel, getNumberOfPes, getProcessingCost, getRequiredFiles, getReservationId, getResourceById, getResourceId, getResourceName, getStatus, getStatusString, getSubmissionTime, getSubmissionTime, getUserId, getUtilizationModelBw, getUtilizationModelCpu, getUtilizationModelRam, getUtilizationOfBw, getUtilizationOfCpu, getUtilizationOfRam, getVmId, getWaitingTime, getWallClockTime, getWallClockTime, hasReserved, isFinished, requiresFiles, setClassType, setCloudletFinishedSoFar, setCloudletLength, setCloudletStatus, setExecParam, setExecStartTime, setNetServiceLevel, setNumberOfPes, setRequiredFiles, setReservationId, setResourceParameter, setResourceParameter, setSubmissionTime, setUserId, setUtilizationModelBw, setUtilizationModelCpu, setUtilizationModelRam, setVmId, write`
    - ### Methods inherited from class java.lang.Object

      `clone, equals, finalize, getClass, hashCode, notify, notifyAll, toString, wait, wait, wait`

- - ### Field Detail


    - #### submittime

      ```
      public double submittime
      ```


    - #### finishtime

      ```
      public double finishtime
      ```


    - #### exetime

      ```
      public double exetime
      ```


    - #### numStage

      ```
      public double numStage
      ```


    - #### currStagenum

      ```
      public int currStagenum
      ```


    - #### timetostartStage

      ```
      public double timetostartStage
      ```


    - #### timespentInStage

      ```
      public double timespentInStage
      ```


    - #### timeCommunicate

      ```
      public java.util.Map<java.lang.Double,HostPacket> timeCommunicate
      ```


    - #### stages

      ```
      public java.util.ArrayList<TaskStage> stages
      ```


    - #### starttime

      ```
      public double starttime
      ```
  - ### Constructor Detail


    - #### NetworkCloudlet

      ```
      public NetworkCloudlet(int cloudletId,
                     long cloudletLength,
                     int pesNumber,
                     long cloudletFileSize,
                     long cloudletOutputSize,
                     long memory,
                     UtilizationModel utilizationModelCpu,
                     UtilizationModel utilizationModelRam,
                     UtilizationModel utilizationModelBw)
      ```
  - ### Method Detail


    - #### compareTo

      ```
      public int compareTo(java.lang.Object arg0)
      ```

      **Specified by:**
      :   `compareTo` in interface `java.lang.Comparable<java.lang.Object>`


    - #### getSubmittime

      ```
      public double getSubmittime()
      ```


- Overview
- Package
- Class
- Use
- Tree
- Deprecated
- Index
- Help

- Prev Class
- Next Class

- Frames
- No Frames

- All Classes

- Summary:
- Nested |
- Field |
- Constr |
- Method

- Detail:
- Field |
- Constr |
- Method
